# Old but gold: an historical perspective of wet mount microscopy and its current role for the diagnosis of vaginitis

**DOI:** 10.3389/frph.2026.1755906

**Published:** 2026-02-05

**Authors:** Lorenzo Agoni

**Affiliations:** Unit of Obstetrics and Gynecology, Fondazione Poliambulanza Istituto Ospedaliero, Brescia, Italy

**Keywords:** aerobic vaginitis, bacterial vaginosis, cytolytic vaginosis, history of vaginitis, trichomoniasis, vaginal infections, vulvovaginal candidiasis, wet mount microscopy

## Abstract

Since the invention of the microscope, physicians and gynecologists have utilized wet mount microscopy of vaginal fluids. A significant milestone was the discovery of Trichomonas vaginalis by Alfred François Donné in 1836. As the century progressed, research by Albert Döderlein shifted focus to the presence of lactobacilli. In the early 1920s, Christine Marie Berkhout provided a detailed description of the fungus Candida. For many years, understanding the microbiology of vaginal fluids in health and disease played a crucial role in diagnosing vaginitis and sexually transmitted infections. The development of culturing techniques on Petri dishes and later molecular biology methods, which became widespread and commercially accessible, offered more accurate diagnostic options, leading to the gradual decline of office microscopy. In this perspective article, we explore the advantages of maintaining office microscopy as a crucial component of gynecological examinations at point of care, especially for diagnosing vaginitis.

## 1 Introduction

Vaginitis is often regarded as a minor health issue in women's lives, perceived merely as a source of discomfort that can be easily managed. However, studies are unanimously recognizing that vaginitis is the most common reason why patients seek gynecological care (1–4). Moreover, it can present significant diagnostic and therapeutic challenges, particularly when it recurs after appropriate treatment (5–8).

In terms of diagnosis, vaginitis can be classified into different types based on the pathogens involved. We distinguish five major and more common types of vaginitis (1): candidiasis, bacterial vaginosis, aerobic vaginitis, trichomoniasis, and cytolytic vaginosis, each of which will be discussed in a dedicated section. The treatment for vaginitis varies depending on the type, making accurate diagnosis crucial for prescribing the most appropriate therapy.

When vaginitis recurs more than three to four times a year, it is referred to as “recurrent vaginitis.” It is estimated that approximately 75% of women experience acute candidiasis at some point in their lives, while around 5%–10% will experience it recurrently (9). Bacterial vaginosis recurrence can be as high as 50%–80% of cases (10). Treatment for recurrent vaginal affections focuses on reducing the frequency, severity, and number of recurrences. However, managing recurrent vaginitis/vaginosis poses significant challenges, as pathogens often develop resistance to prolonged antibiotic use. Several treatment regimens have been developed to balance efficacy while minimizing the risk of antibiotic resistance (11–13).

Beyond being a source of discomfort, vaginitis can represent a significant health issue and a risk factor for acquiring sexually transmitted infections (STIs), such as gonorrhea and chlamydia (14–17). These STIs may present with severe symptoms or can be asymptomatic, yet they carry a risk of developing pelvic inflammatory disease (PID), which can lead to female infertility (18). Similarly, bacterial vaginosis increases the risk of acquiring HIV in those countries where this infection is endemic (19–21). Additionally, human papillomavirus (HPV) infection is linked to bacterial vaginosis and is a well-known risk factor for cervical cancer (22, 23).

Certain types of vaginitis also affect reproductive outcomes. For example, bacterial vaginosis may increase the risk of preterm delivery, defined as delivery before the 37th week of gestation, as elaborated in a subsequent section of this paper (24, 25).

Therefore, vaginitis can no longer be regarded merely as a source of discomfort. Gynecologists and other healthcare providers encountering symptoms of vaginitis in their patients should accurately diagnose the specific type to prescribe the appropriate treatment. A multitude of diagnostic tools is now available to ensure the most accurate diagnosis. While microscopy was historically the most common technique for diagnosing vaginitis, today, swabs for culturing pathogens on Petri dishes or for molecular methods such as nucleic acid amplification tests (NAATs) are more widely used. However, not every method is suitable for diagnosing specific types of vaginitis, and accuracy may vary based on the method used and the pathogen involved (26). Guidelines have been established to assist physicians in selecting the most appropriate test based on the patient's symptoms and clinical suspicion (27–30).

The purpose of this article is to show how microscopy shaped the field of vaginal infections during history and can still, despite other technological advancements, support gynecologists in achieving an accurate diagnosis of vaginitis directly during the office visit. Surprisingly, microscopy may still outperform more recent techniques when considering time-to-diagnosis and affordability. Wet mount microscopy remains the gold standard for diagnosing specific types of vaginitis, such as aerobic vaginitis and cytolytic vaginosis. Additionally, it provides quick and useful results for diagnosing candidiasis, trichomoniasis, and bacterial vaginosis, although it is less sensitive than other methods such as culturing or nucleic acid amplification tests (NAATs). In this article, we will explain why gynecologists should not abandon wet mount microscopy but instead routinely use it during the visits.

## 2 Different types of vaginitis

Vaginitis can be defined as the inflammation of the vaginal mucosa, often correlated to symptoms such as unusual vaginal discharge, odor, irritation, itching, or burning sensations. This condition may be caused by bacterial, yeast or protozoal infections, as well as non-infectious factors such as irritants, allergies, or hormonal changes (26). Understanding the underlying cause is crucial for effective management.

Historically, vaginitis has been an elusive entity. Vaginal discharge may have various characteristics such as quantity, color, smell and may be correlated with symptoms such as itching or burning (31, 32). The advent of the microscope changed the perspective on how to study vaginitis and explore potential etiologic causes for its occurrence. The first to observe live microorganisms with a more potent version of the first instrument invented in 1590, now capable of a 200× power magnification, was Antonie van Leeuwenhoek, in 1676 (33). Since then, the era of microscopic examination of biological entities such as microorganisms and human cells and tissues begun.

Among the various types of vaginitis, bacterial vaginosis, with prevalence of 20%–30% among the female population worldwide, is probably the most common condition (8). It is defined by the overgrowth of Gardnerella vaginalis and other bacteria, collectively called “Bacterial Vaginosis Associated Bacteria (BVAB)”, which include Fannyhessea vaginae (formerly known as Atopobium vaginae) and other less characterized bacteria (34, 35).

Bacterial vaginosis is typically correlated to a thin grayish vaginal discharge with fishy odor, which may impact on sexual life (36), but no symptoms of itching, burning or pain during intercourse. Bacterial vaginosis is often recurrent, regardless initial successful treatment (8). At wet mount microscopic examination bacterial vaginosis appears as a fuzzy bacterial background, in which single bacterial cells are difficult to distinguish from one another, often depleted of lactobacilli, which culminates in the detection of the so called “clue cells” which are vaginal epithelial cells showing adherent bacilli on their surface, typically of Gardnerella vaginalis species (37).

Another common vaginitis is correlated to yeast infections, mostly by Candida albicans (38). Less common Candida species, such as C. glabrata, C. krusei, C. parapsilosis, C. tropicalis and others, may be involved. Vaginal candidiasis has and approximate prevalence of 10%–20% in women worldwide. It often involves the vulvar area, thus it is better defined as vulvovaginal candidiasis, and it usually manifests with a white, thick, curd-like odorless discharge, itching, vulvar erythema and dyspareunia (39). Approximately 75% of women experience at least one episode of vulvovaginal candidiasis in their life and 10% of them have frequent recurrences, occurring at least 3–4 times a year (40). At wet mount microscopy Candida species are evident by detection of either or both hyphae or blastospores, which constitutes the cells of Candida with their typical morphology. Hyphae are elongated often branched formations that remembers the branching of trees; blastospores are small oval cells, often characterized by a protruding bud, which can be elongated, in which case this are called “pseudo-hyphae”. The presence of hyphae or pseudo-hyphae is diagnostic for Candida albicans, as other species of Candida typically do not form hyphae and remain at the stage of blastospores (41, 42).

A third common type of vaginitis is aerobic vaginitis, which shows prevalence of approximately 7%–12% in women worldwide (43). It is sustained by aerobic (facultative anaerobic), bacteria such as Escherichia coli, Enterococcus faecalis and Streptococcus agalactiae, migrating from the intestine to the vagina. Typically, symptoms include a yellowish or greenish mucoid discharge, with foul odor, vaginal inflammation, often vaginal desquamation or atrophy, and painful intercourse. At wet mount microscopy aerobic vaginitis is characterized by a background of bacteria which can be clearly seen a single cells, thus the background is not “fuzzy” as in bacterial vaginosis. Both cocci and bacilli can be detected and lactobacilli are often depleted or absent. Typically, white blood cells (WBC) are identified, often polymorphonucleated neutrophils (PMN). Moreover, vaginal epithelium parabasal cells are present in higher amount than expected, also in pre-menopausal women, which is a sign of desquamation of the epithelium, typical of aerobic vaginitis. In fact, when PMN and parabasal cells are prevalent the condition is called “desquamative inflammatory vaginitis” (DIV) (43, 44).

A less common cause of vaginitis is secondary to a protozoal microorganism: Trichomonas vaginalis. This is considered an actual sexually transmitted infection (STI) and it can affect the male partner as well, both asymptomatically and with typical symptoms of urethritis. Although the mean global prevalence of trichomoniasis is approximately 8%, it greatly varies across different part of the world, being more common in Africa (12%) and less common in Europe (3%) (45).

Trichomoniasis usually presents with a greenish or yellowish frothy discharge, and speculum examination may reveal cervical erythema with petechiae, the so called “strawberry-like cervicitis” (46). At the wet mount microscopy the protozoal cells can be easily spotted for their motility, enabled by a set of five flagella, and peculiar oval shape characterized by a prominent nucleus (47).

We conclude the list of the five most typical vaginitis with the para-physiological condition of cytolytic vaginosis, which is characterized by vaginal cell cytolysis due to overgrowth and overactivity of resident normal lactobacilli (48). The prevalence of cytolytic vaginosis is debated as often it is asymptomatic. On average, it has been estimated a global prevalence of 5% in women (49). When symptomatic, complaints can include vulvovaginal itching, burning, and a thick, white, clumpy odorless discharge, and dyspareunia. At wet mount microscopy this condition is characterized by evident cytolysis which is determined for the presence of cytoplasmatic debris and nuclei without their surrounding cytoplasm, the so called “naked” nuclei. Typically, lactobacilli are prevalent and over-abundant (50, 51).

The most common vaginitis with signs and symptoms and wet mount microscopy findings are summarized in Table 1.

**Table 1:** Characteristics of most common vaginal conditions.

Condition	Etiological agents	Signs and symptoms	Wet mount microscopy findings
Bacterial vaginosis	G. vaginalis and BV related bacteria	Thin grayish vaginal discharge with fishy odor, no symptoms of itching, burning or dyspareunia, elevated pH, positive whiff test	Clue cells, fuzzy background flora, depleted lactobacilli, none or sporadic leukocytes
Candidiasis (C. albicans)	C. albicans	White, thick, curd-like odorless discharge, itching, vulvar erythema, dyspareunia, pH may vary, negative whiff test	Hyphae, pseudohyphae, blastospores, often leukocytes
Candidiasis (non albicans species)	C. glabrata, C. krusei, C. parapsilosis, C. tropicalis, and others	White, thick, curd-like odorless discharge, itching, vulvar erythema, dyspareunia, pH may vary, vegetive whiff test	Blastospores, often leukocytes
Aerobic vaginitis	Escherichia coli, Enterococcus faecalis and Streptococcus agalactiae, and others aerobic bacteria from the intestine	Yellowish or greenish mucoid discharge, with foul odor, vaginal inflammation, often vaginal desquamation or atrophy, dyspareunia, elevated pH, negative whiff test	Cocci or coliform bacilli, depleted lactobacilli, often leukocytes, often parabasal cells
Trichomoniasis	T. vaginalis	Greenish or yellowish frothy discharge, “strawberry-like cervicitis”, elevated pH, negative whiff test	Motile protozoal cells, leukocytes
Cytolytic vaginosis	Lactobacilli	Itching, burning, and a thick, white, clumpy odorless discharge, dyspareunia, low pH, negative whiff test	Overabundant lactobacilli, “naked” nuclei, cytoplasmatic debris, none or sporadic leukocytes

This table summarizes the most common vaginal conditions listed in column 1. Typical etiological agents are listed in column 2. Signs and symptoms are highlighted in column 3. Wet mount microscopy findings are shown in column 4. This table can be used to navigate through signs, symptoms and findings for efficient differential diagnosis.

Another physiological or para-physiological condition is vulvovaginal atrophy, which can affect a significant percentage of the post-menopausal female population, up to 50% in late postmenopausal state (27). The most common symptoms include vaginal dryness, burning, itching, and painful intercourse, sometimes accompanied with postcoital bleeding. Similar symptoms can be experienced in women under progestin state both naturally, such as during postpartum and puerperium, or secondary to hormonal contraceptive use (52). At wet mount microscopy an unusual abundance of parabasal cells is seen. These cells may be present due to hormonal depletion, in post-menopause, or progesterone drive, in post-partum or during hormonal contraceptive use. PMNs and bacteria other than lactobacilli should not be apparent, otherwise the diagnosis would more likely be of aerobic vaginitis (53).

Other cases of pathological, often purulent, vaginal discharge are associated to STIs such as syphilis or gonorrhea (30, 54, 55). At wet mount microscopy an extensive background of PMNs can be seen. Also, the specific pathogens can be detected: Treponema pallidum, which is the etiological agent of syphilis, can be detected as spiraliform bacillus, 6–15 µm long and 0.1–0.2 µm wide on average, characterized by its motility allowed by a set of endoflagella, which are flagella located right underneath the bacterial wall. Because of is small dimension and thinness it may be difficult to detect at the microscopic examination. However, its motility is characteristic and may allow it to be visualized, particularly with dark field (56).

Discharge from Chlamydia trachomatis, Mycoplasma or Ureaplasma may be less typical, and infections are often completely asymptomatic, although dysuria or strangury may be present (57, 58). At wet mount microscopy the presence of Chlamydia, Mycoplasma or Ureaplasma may pass unnoticed as these bacteria are extremely small, approximately 0.2–0.4 µm in diameter, and they often parasite vaginal epithelial cell intracellularly. Because of this, intracellular inclusions may be noticed in vaginal epithelial cells during the microscopic analysis, especially for Chlamydia (59).

Unusual causes of vaginal complaints include foreign body retention (60) cervical cancer (60), Sjögren syndrome (61), irritative or allergic reactions (62). At wet mount microscopy may show morphologic alterations of epithelial cells, especially in case of cancer, and inflammation with a homogenous layer of PMNs. In these cases various types of vaginitis may overlap and differential diagnosis is crucial.

## 3 Milestones of diagnosis

In the beginning there was only chaos. Without knowing the etiologies that differentiate the various vaginitis, these conditions were regarded as a unicum, often framed into traditional beliefs, such as the imbalance of body energy and fluids.

**Figure 1:**
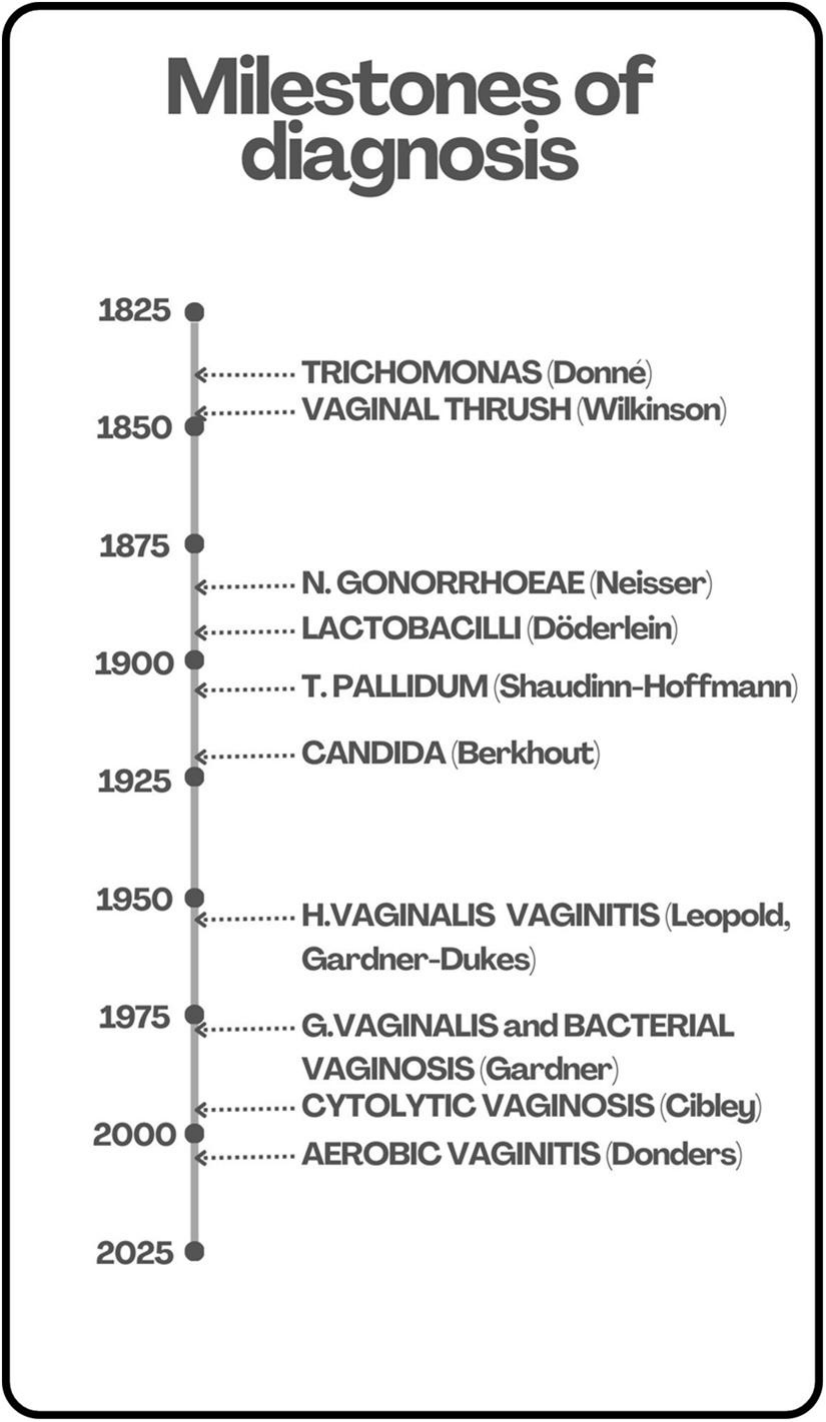
Milestones of diagnosis with microscopy. The discoveries and definitions of vaginitis are shown on a timeline. In 1836, Donné (63) discovered Trichomonas vaginalis from vaginal discharge of a symptomatic patient. In 1849, Wilkinson (66) defined vaginal thrush by identifying a vaginal yeast similar to that previously described in oral thrush. Later, in 1923, this was renamed as Candida by Berkhout (67). In 1879, Neisser (68) discovered Neisseria gonorrhoeae by examining urethral purulent secretions in a symptomatic patient. In 1892, Döderlein (70, 71) defined the vaginal bacilli, later identified as lactobacilli, as the normal vaginal flora. In 1905, Schaudinn and Hoffmann (72) discovered the etiological agent of syphilis, Treponema pallidum, by examining a fresh preparation of material taken from a vulvar lesion. In 1953, Leopold discovered Hemophilus vaginalis. In 1955, Gardner and Dukes (75, 76) defined that Hemophilus vaginalis was the causative agent of the then so called “non-specific vaginitis”. In 1980 Hemophilus vaginalis was renamed as Gardnerella vaginalis (77) and the term “non-specific vaginitis” replaced with “bacterial vaginosis” (78), because inflammation was irrelevant for the disease. In 1991, Cibley and Cibley (80), a father-son team, defined the “cytolytic vaginosis”. In 2002, Donders (44) defined the criteria for “aerobic vaginitis”. Further details in the text.

Then the modern microscope was invented, and it shed light into the chaos (Figure 1). Alfred Francois Donné (1801–1878) (63) a French scientist, was a pioneer in microscopy, and he applied this technique to various field of medicine, from microbiology to hematology. He is credited for being the first to identify Trichomonas vaginalis into the vaginal secretions of a symptomatic patient, in 1836. He was also the first to take photographs (daguerreotype) of the microscopy findings, thanks to the photoelectric microscope of his invention.

Thanks to his efforts and the following characterization of the protozoa we now know well what T. vaginalis is. It can be described as an ovoidal motile microorganism, 15–20 μm long and 8–10 μm wide, characterized by four anterior flagella, a fifth flagellum recurring back on its body, an undulating membrane located on both sides, and an axostyle which traverses its body emerging back as a spike (64). This microorganism typically infects the urogenital tract of both males and females and it is easily sexually transmitted. Because its presence is far from being a normal occurrence of the local microbiome, it is considered an actual STI that requires systemic medical treatment in both partners (65).

In 1849, Stuart Wilkinson, a Scottish physician, identified, at the microscope, a vaginal yeast similar to that previously described in oral thrush (66). The current name of the yest as Candida was first published in 1923 in the doctoral thesis of the Dutch botanist and mycologist Christine Marie Berkhout (1893–1932) (67).

In 1879, Albert Ludwig Sigesmund Neisser (1855–1916) (68), a German physician, discovered Neisseria gonorrhoeae by examining urethral purulent secretions in a symptomatic patient. The bacteria are round, approximately 0.6-1 μm in diameter, with the typical appearance in pairs of cells, thus they are defined as diplococci.

Common symptoms of gonorrhea in women include unusual vaginal discharge, which can assume various presentations from thin to purulent, dysuria or strangury, and dyspareunia. However, gonorrhea tends to be more commonly symptomatic in males than in females, in which the infection can pass unnoticed. The World Health Organization (WHO) estimated a global prevalence of 0.9% in women and 0.7% in men for this STI, but precise prevalence may vary in different regions of the world, being higher in Africa and lower in Europe (69).

A significant step forward for the comprehension of the vaginal microbiology occurred in 1892 when Albert Sigmund Gustav Döderlein (1860–1941) (70), a German gynecologist, begun to study the thin and elongated bacilli that were so easily found in the vaginal fluid of asymptomatic women. He realized that these bacilli constituted the normal vaginal flora and their absence in pregnancy and postpartum was a risk factor for the development of the puerperal fever, which could quickly turn to the worse and lead to death (71)

In 1905, Fritz Schaudinn, a German zoologist, and Erich Hoffmann, a German dermatologist, working together in Berlin, discovered the etiological agent of syphilis, Treponema pallidum, by examining a fresh preparation of material taken from a vulvar lesion of a patient (72). This bacterium has the peculiar shape of a spiral, from which the name of the phylum “Spirochaetes”. It is approximately 6–15 μm long and 0.1–0.2 μm wide. It is easily recognized in wet mount microscopy, particularly in dark field, because of its motility.

Treponema pallidum does not typically cause vaginitis, but its genital painless ulcers can be easily identified. Rarely, vaginal or cervical lesions, which may be clinically mistaken for cancerogenic ulcers, can cause unusual vaginal discharge in which the bacteria can be easily recognized at the microscope (73).

Syphilis is an STI with global prevalence of approximately 0.6%, with higher prevalence in low-income countries. Although limited in prevalence, it is re-emerging in the past decade as a global concern (74).

Thus, in the first decades of 20th century, a few diagnoses of vaginitis were available to the physicians: mostly vaginal candidiasis, but also trichomoniasis and STIs such as gonorrhea or syphilis. Frequently, vaginal complaints were not associated to none of such conditions. At that time this was called “non-specific vaginitis”.

In 1955, Herman L. Gardner (1912–1982) and Charles D. Dukes, from Texas (USA), proposed that the then recently discovered bacterium Haemophilus vaginalis was the etiological cause of “non-specific vaginitis”, which was then called “Haemophilus vaginalis vaginitis” (75). In 1959, they also defined that this condition was not associated to vaginal inflammation and, thus, they proposed that “vaginitis” was not an appropriate definition (76). Later, in 1980, Haemophilus vaginalis was officially renamed to Gardnerella vaginalis (77), to honor who spent his all life in characterizing this bacterium and the implications of its presence and overgrowth in the vagina. Also, the term “bacterial vaginosis” was coined to describe this condition of overgrowth of G. vaginalis and other anaerobic bacteria, and it rapidly gained wide acceptance in the scientific field (78).

The cornerstones for diagnosis of bacterial vaginosis were a thin grayish vaginal discharge, high vaginal pH (>4.5), a fishy odor, which could be amplified by adding a droplet of 10% KOH solution to the vaginal secretion placed on a microscopy slide, and the presence of the so-called “clue cells” at the microscopic examination. The “clue cells” are squamous vaginal epithelial cells that are covered with the G. vaginalis rods, which are highly adhesive to the mucosa. These criteria were proposed by Richard Amsel, an American gynecologist, in 1983 and became widely used for office diagnosis of bacterial vaginosis (79).

Nonetheless, bacterial vaginosis could not explain everything of the previously called “non-specific vaginitis”. There were occurrences of vaginitis characterized by inflammation, thus with presence of leucocytes at the microscopic examination, absence of clue cells, heavy cocci or rods presence, often squamous cell atrophy, without fishy odor, more likely a foul odor, and a mucous yellowish vaginal discharge. This condition often has a low-intermediate score at the Amsel criteria, thus excluding the diagnosis of bacterial vaginosis. Moreover, G. vaginalis did not seem to be involved. Instead, bacteria such as Escherichia coli, Enterococcus, Streptococcus, Klebsiella and other facultative anaerobic bacteria were often present. For this condition Gilbert Donders, a Belgian gynecologist, coined the term of “aerobic vaginitis” in 2002 (44). The criteria for diagnose aerobic vaginitis rely on presence/absence of lactobacilli, presence/absence of background flora, such as cocci or rods, presence of leukocytes and activated leukocytes, and presence of parabasal cells. Thus, it is an exquisite office microscopy diagnosis (43).

We close now the list of vaginitis with the definition of “cytolytic vaginosis” coined in 1991 by Leonard and Laurence Cibley, a father-son physician team from Boston (MA—SA) (80). The criteria for diagnosis include the absence of Trichomonas, Gardnerella and Candida, an increased number of lactobacilli, scarcity of leukocytes and evidence of cytolysis with bare nuclei and cellular debris. Vaginal discharge may be whiteish, frothy or dense, but always with a low pH (3.5–4.0). The patient may also experience itching, burning, dysuria and dyspareunia. This condition is often underestimated, because its symptoms overlap with other vaginitis symptoms and may also be asymptomatic, and may be more prevalent than previously suspected (81).

As described so far, the discoveries of etiological factors of vaginitis were essentially achieved by microscopy examination. Besides wet mount microscopy, numerous staining techniques arose in those years and helped more accurate detection and diagnosis. We can just mention the staining by the Danish bacteriologist Hans Christian Gram (1853–1938) (82) or the German chemist Gustav Giemsa (1867–1948) (83), which were invented in 1884 and 1902, respectively, and are still incredibly useful for the study of alcohol fixed microorganisms on microscopy slides. Microscopy remained an important technique throughout the decades. At the same time, though, culturing methods became increasingly available and became useful resources for accurate diagnosis.

These techniques allowed the study of the biochemical properties of bacteria and resulted very useful in define nutritional needs and enzymatic capabilities to differentiate bacteria that may look similar, or even identical, under the microscope (84). Moreover, bacterial culturing allows the study of bacterial susceptibility or resistance to antibiotics, which leads to choose the most appropriate therapy for each specific patient (85).

However, to date, is has been estimated that only a small fraction of bacterial species can grow in a Petri dish in the laboratory (86). This became clear with the advent of Polymerase Chain Reaction (PCR) and even more with the most recent advancement of the Next Generation Sequencing (NGS) technique, available since 2004, which allowed to identify even those bacterial species that cannot grow in culture, by amplifying sequences of their DNA. Collectively, we can call these nucleic acid amplification tests (NAATs).

The advent of new techniques did not make the previous ones disappear. Thus, now wet mount microscopy, Gram staining, culturing, and NAATs techniques they all coexist and are available in the clinical setting. In conclusion, the clinician can currently choose among a few different techniques to address his patient's symptoms and accurately identify the specific type of vaginitis. Each technique shows advantages and disadvantages and may be more or less useful for specific diagnoses. We will address this issue in later sections of this paper.

## 4 Gynecological visit for the diagnosis of vaginitis

Different vaginitis typically have different clinical presentations. For instance, pruritus and thick white discharge recall candidiasis, thin fishy odor greyish discharge recalls bacterial vaginosis, strawberry cervicitis recalls trichomoniasis, and a mucoid yellowish foul odor discharge recalls aerobic vaginitis. Also, an accurate anamnesis does help in addressing the problem and better understanding the etiology. For example, we would expect dyspareunia in some conditions, such as candidiasis, but not in others, such as bacterial vaginosis. Many gynecologists rely on this knowledge to empirically define a clinical diagnosis and prescribe medications (87).

Although different vaginitis typically have different clinical presentations, symptoms may overlap leading to challenging diagnosis. Moreover, symptoms may be subtle, or vaginitis can be mixed, for including different etiologies. Therefore, the gynecological visit alone is not considered the gold standard for diagnosis vaginitis by agencies such as ISSVD (27), CDC (30), or IUSTI/WHO (29).

A few point of care (POC) tests are available to help the gynecologist in defining the most accurate diagnosis. The first POC test that can be easily performed during the pelvic exam is the pH testing. Paper strips that can measure pH are widely available. A high pH (>5.0) is more often found in bacterial vaginosis, trichomoniasis and aerobic vaginitis, while a low pH (<4.0) is more typical of cytolytic vaginosis (88). The pH does not really help with candidiasis as Candida can be found at a wide range of pH.

The “sniff” test, which is the perception of the discharge odor on the speculum, can be considered, if positive, as appropriate for the diagnosis of bacterial vaginosis. The fishy odor typical of bacterial vaginosis can be evoked also by adding a droplet of 10% KOH solution on the glass slide on which the vaginal secretion has been previously smeared, the so called “whiff” test (89).

The wet mount microscopy remains the most complete POC exam that a gynecologist can perform on vaginal secretions. We will describe that in detail in the next section of this paper.

Besides POC tests, gynecologists can take advantage of laboratory exams such as Gram staining, bacterial culturing, and DNA detection by NAATs. In all these cases it takes just the time of a vaginal swab to collect the sample but then several days will be required to have the laboratory results back. Many gynecologists would consider this a trade-off for accurate diagnosis.

Gram staining has several interesting features that are useful for diagnoses. Firstly, it can identify both lactobacilli and leukocytes which are not detected with other methods such as culturing or PCR. Also, it can visualize “clue cells” for the diagnosis of bacterial vaginosis. In fact, a specific score based on Gram staining was proposed by Robert P. Nugent in 1991 (90). The score is based on the presence of lactobacilli, which are Gram-positive, Gardnerella and related species, which are Gram-variable, and presence of curved rods, such as Mobiluncus, which is Gram-variable. The Nugent score is considered more reliable than the Amsel's criteria for the diagnosis of bacterial vaginosis. Comparative cross-sectional studies show that when compared to Nugent scoring system, Amsel's criteria had sensitivity of 75%, specificity of 95%, positive predictive value of 90% and negative predictive value of 86% (91).

Moreover, Gram staining can identify bacilli and cocci species such as Staphylococcus and Streptococcus species, which are Gram-positive cocci arranged is groups or long lines, respectively, Neisseria gonorrheae, which is a Gram-negative diplococcus, Enterocuccus species, which are Gram-positive cocci often arranged in pairs (diplococci) or short chains, as well as rods such as Escherichia coli, which is a Gram-negative bacillus, similar to Klebsiella, which is also a Gram-negative rod but it tends to be larger than E. coli.

Gram staining can also diagnose trichomoniasis. In fact, the T. vaginalis cells are more easily identified by their motility, which is lost during the process of fixation and staining, thus making the diagnosis more difficult (92).

Fungal hyphae and pseudo-hyphae can be easily identified with Gram staining as Gram-positive filaments. In fact, blastospores visualization can be more challenging thus the diagnose of non-albicans Candida species can be more difficult (93).

Microscopy has the limitations of being subjective, to some extent, experience-dependent, semi-quantitative at best, and unable to accurately identify many microorganisms, particularly intracellular pathogens such as Chlamydia, Mycoplasma, and Ureaplasma (94).

Culturing methods can overcome many of such limitations. By using selective medium, the microorganisms will grow colonies that can be further analyzed by biochemical methods to reach an accurate diagnosis. An advantage of culturing is that it can detect the selected microorganisms even when their presence in the sample is at low level and it may be pass underdetected at the microscopy examination. On the other hand, culturing is time consuming and several days are needed to reach the diagnosis. Also, many microorganisms require specific culturing medium, thus complicating the overall process. Some microorganisms are difficult to grow in culture, such as Mycoplasma, other just cannot grow, such as Chlamydia (95–97).

An interesting advantage of culturing consists in semi-quantitative analysis, by determining the number of colonies that can grow from the sample. Moreover, with culturing we can test different antibiotics against the identified pathogen. This helps clinicians in deciding which antibiotic to prescribe in the specific patient (98).

PCR has several advantages over microscopy and culturing. For instance, PCR relies on an automated machine process which avoid most of the human variability and error that can occur with microscopy or culturing. It is more sensitive than microscopy and culturing as it can detect the targeted microorganisms even when present at very small amount. It is more specific than microscopy and culturing because it directly targets the DNA sequence of the microorganisms, and it allows a faster diagnosis (99–101).

Another clear advantage of PCR is that it can detect also microorganisms that do not grow well or at all in culture, such as Chlamydia, Mycoplasma and Ureaplasma. On the other hand, it can detect only what it targets. In other words, a PCR reaction must be set for each microorganism of choice. For example, Sneathia is a Gram-negative coccobacillus often found in bacterial vaginosis, but it was isolated only in 2002, from the amniotic fluid of an intrauterine fetal loss, because it does not grow in culture. Now, with PCR, it can be detected also in bacterial vaginosis, given that we use the PCR probes designed for it (102).

A significant leap forward in the comprehension of microbial environments is constituted by the advent of NGS techniques. With a single NGS analysis one can detect virtually all the microbial species that are present in the sample (103, 104). Regardless the incredible level of detail that can be reached with NGS, this technology cannot detect leukocytes or cytolysis, which are important details for some subtypes of vaginitis as above described.

In conclusion, regardless the technical advancements, there is no modern technique capable to singlehandedly diagnose every type of vaginitis like wet mount microscopy used to do in the early days.

## 5 Office wet mount microscopy

A phase contrast 400× magnification microscope constitutes the most appropriate instrument for observing cells and bacteria. Digital camera microscopes are also commercially available and allow to take photographs of the samples under magnification to document the evidence.

**Figure 2:**
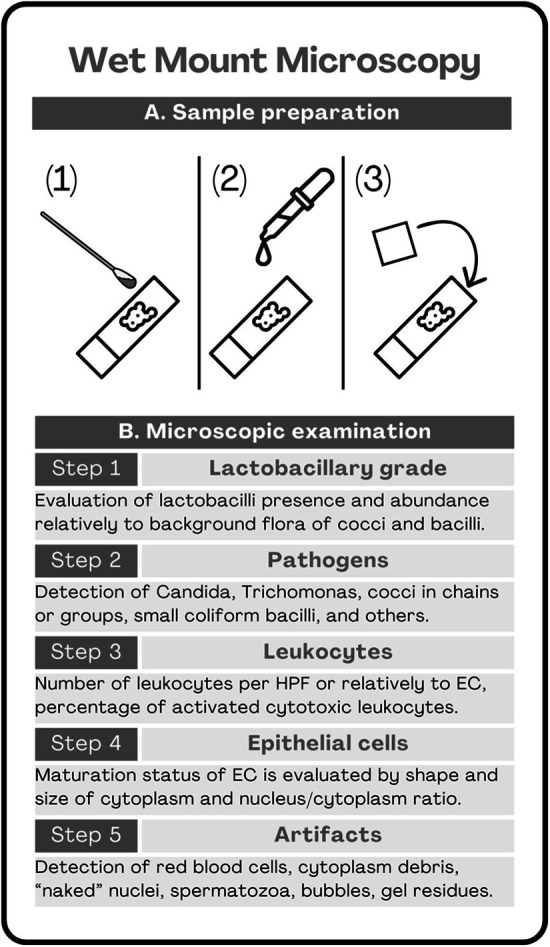
Wet mount microscopy, preparation and examination. In the upper panel **(A)** the steps for sample preparation are shown (105, 106): (1) the vaginal swab is smeared on a microscopy glass slide, (2) a droplet of saline solution is added, (3) a coverslip is laid on top of the droplet. In the lower panel **(B)** the 5 steps for proper sample examination are explained. Step 1 scores the presence of lactobacilli with the so-called “Lactobacillary grade” (107): in grade I only lactobacilli are visible, in grade IIa there is a dominance of lactobacilli over background flora, in grade IIb there is a dominance of background flora over lactobacilli, in grade III background flora is absolute and no lactocabilli can be detected. Step 2 is dedicated to the identification of the pathogens (108–112). Step 3 scored the presence of leukocytes, their number per HPF, per EC, and proportion of activated cytotoxic leukocytes (113). Step 4 considers size, shape and cytoplasm/nucleus ratio of EC to determine their maturation level and distinguish superficial layer cells from intermediate layer calls from parabasl layer cells. The number and proportion of parabasal cells is relevant to aerobic vaginitis score (43, 44). Step 5 lists the presence of artifacts such as red blood cells, cytolysis or amorphous material. Further details in the text. Legend: HPF = high power fields (400× magnification), EC = epithelial cells.

From a technical standpoint preparing a slide for wet mount microscopy is quick and easy (Figure 2A): a drop of vaginal secretion is collected through a swab during the speculum examination and smeared on a glass slide. Then a drop of saline solution is put on the slide, and a coverslip is carefully placed on top. The sample is then ready for analysis at the microscope (105, 106).

Classically, the analysis is performed in five steps (Figure 2B). The first step is to evaluate the presence and abundancy of lactobacilli as compared to other bacteria in the observation fields. A four-tire “lactobacillary grade (LBG)” was proposed to categorize this item. While LBG-I is correlated to the exclusive presence of lactobacilli, on the other extreme, LBG-III, scarce to no presence of lactobacilli with great abundance of non-lactobaclliary bacteria. In the middle, two intermediate situations with LBG-IIa with predominance of lactobacilli over a non-lactobacillary background, and with LBG-IIb with the predominance of non-lactobacillary background over lactobacilli presence (107).

The second step is dedicated to the detection of pathogens. Phase contrast microscopy allows the identification of the presence of cocci and rods, although it cannot determine the exact species (108). However, some bacteria have peculiar appearance or presentations allowing a more accurate identification. For instance, Mobiluncus is visualized as a curved “comma-like” rod (109), Streptococcus tends to form cocci chains, Staphylococcus forms groups of cocci, Enterococcus tends to be visualized as diplococcus or forming short chains, and Neisseria is always detected as diplococcus (110). Other microorganisms can be easily visualized for their motility, Trichomonas (111), for instance, but also Escherichia coli, which is not always easily seen as motile, Treponema pallidum or other Spirochetae. Yeast cells can easily be detected for their elongated hyphae or pseudo-hyphae, typically Candida albicans. Blastopores, which can include non-albicans Candida species, are more subtle to be detected as they look like small ovoidal bright cells (112).

The third step focuses on the presence of leukocytes, which is a sign of inflammation and may be correlated to the patient's complaints such as burning or dyspareunia. The number of leukocytes per high power field (HPF) is determined as well as the ratio with the number of epithelial cells. Also, the presence of activated cytotoxic leukocytes is evaluated. These numbers are often used for scoring, especially for aerobic vaginitis (43, 44).

In the fourth step the epithelial cells are examined. The ratio between superficial, intermediate and parabasal cells is important to determine the maturation status of the epithelium. Particularly, the presence and abundance of parabasal cells is included in the scoring system for aerobic vaginitis (44). A significant number of parabasal cells may be detected for two reasons: vaginal atrophy in postmenopausal women, desquamative vaginitis in premenopausal women. Moreover, the microscopy analysis can detect cytolysis by visualizing bare nuclei or cytoplasmatic debris, typical of cytolytic vaginosis (113).

Finally, in the fifth step other occurrences may be annotated, such as: red blood cells, sperm cells, or artifact from the use of vaginal tablets or lubricant gel residues.

In five quick steps every type of vaginitis can be diagnosed directly during the gynecological visit by wet mount microscopy. Then why wet mount microscopy is not the gold standard for the diagnosis of all kinds of vaginitis? Because sensitivity and specificity may vary depending on the pathogen and other techniques may be more sensitive or specific for certain pathogens (42). We will address these differences in the next section of the paper.

## 6 Contemporary diagnosis of vaginitis

Few international guidelines are available for the diagnosis of vaginitis. The guidelines issued by ISSVD (27), CDC (30), ACOG (28), and IUSTI/WHO (29) it will be here summarized, highlighting strengths, weaknesses and differences.

The gynecological visit alone is not considered the gold standard for diagnosis for vaginitis by none of the guidelines. Signs, symptoms and history are important for a more comprehensive understanding of the clinical situation but alone, although suggestive, they should not be used to define diagnosis.

Vulvovaginal candidiasis can be detected by wet mount microscopy, Gram staining microscopy, yeast culture, and NAATs. The gold standard for the ISSVD guidelines is culturing, while for IUSTI/WHO and CDC guidelines is microscopy. Both are considered valid options for ACOG guidelines. The CDC advice to proceed to yeast culturing when signs and symptoms are coherent with candidiasis but wet mount microscopy is negative. The use of 10% KOH instead of saline solution in wet mount microscopy may increase the detection rate by disrupting cellular material that may obscure the vision of the yeast. The pH detected using pH strips may be within normal range, thus it is not useful for diagnosis. NAATs can be used and are very accurate for the diagnosis of various Candida species. Recently the FDA have approved a few NAAT platforms for clinical use for the diagnosis of candidiasis (114). NAATs, despite being quick and accurate, are still more expensive when compared to the other methods, thus less widely employed (115).

Bacterial vaginosis can be detected by wet mount microscopy and Gram staining microscopy. The former can be used to detect the so called “clue cells” used for the Amsel's criteria (Table 2) of diagnosis that include the quality of the discharge, the evoking of the typical fishy odor by applying 10% KOH, the increase in pH above 4.5. The latter is used to arrange the Nugent score (Table 3) based on the presence of three bacterial morphotypes: Lactobacillus, Gardnerella, and Mobiluncus. Gram staining is also used for the Ison-Hay criteria (116) (Table 4) which still evaluate the three bacterial morphotype just mentioned, but include also cocci morphotypes, which makes this scoring system useful also for the diagnosis of aerobic vaginitis. Gram staining microscopy, either Nugent or Ison-Hay score, are considered the gold standard for bacterial vaginosis diagnosis by ISSVD, IUSTI-WHO, and CDC. For AOCG guidelines both Amsel's criteria and Gram stain are valid for diagnosis. Culturing does not have a role in bacterial vaginosis diagnosis as Gardnerella can be found, in small amounts, even in eubiotic vaginal microbiota. Similarly, for NAATs when used to detect Gardnerella alone. However, NAATs have been evolving fast and a few FDA approved platforms can now detect a panel of bacterial vaginosis associated bacteria besides Gardnerella, thus resulting more accurate in detecting the condition, and useful to the clinician (114).

**Table 2:** Amsel's criteria.

Amsel's criteria
Vaginal discharge: thin, grayish or whiteish
Vaginal pH > 4.5
Fishy odor (“sniff” test or “whiff” test with addition of 10% KOH)
Presence of “clue cells” at wet mount microscopy

These criteria were developed by Amsel to allow the diagnosis of bacterial vaginosis (79). Four clinical criteria should be evaluated, with the support of pH strips, 10% KOH solution, and a microscope, the positivity of a minimum of three criteria is diagnostic for bacterial vaginosis.

**Table 3:** Nugent score.

Score	Lactobacilli (cells/HPF)	Gardnerella (cells/HPF)	Mobiluncus (cells/HPF)
0	>30	0	0
1	5–30	<1	1–5
2	1–4	1–4	>5
3	<1	5–30	–
4	0	>30	–

This scoring system was developed by Nugent to standardize the diagnosis of bacterial vaginosis (90). It is based on detection and grading (average number of cells per high power field—HPF—at 400× magnification) of three different bacterial morphotypes (Lactobacilli, Gardnerella, and Mobiluncus) with gram staining at the microscopy examination. The total score is interpreted as: Normal (0–3), Intermediate (4–6), Bacterial vaginosis (>6).

**Table 4:** Ison-Hay score.

Grade	Classification	Description
0	Normal flora	Epithelial cells with no bacteria
I	Normal flora	Predominance of *Lactobacillus* morphotypes only
II	Intermediate flora	Mixed bacterial morphotypes present, with reduced numbers of *Lactobacillus* morphotypes. *Gardnerella* or *Mobiluncus* morphotypes may also be present.
III	Bacterial vaginosis	Predominantly *Gardnerella* and/or *Mobiluncus* morphotypes. Few or absent *Lactobacillus* morphotypes
IV	Aerobic vaginitis	Gram-positive cocci only, with no lactobacilli

Similarly to the Nugent score, the Ison-Hay score (116) was developed for the diagnosis of bacterial vaginosis and it employes the Gram staining microscopy detection of Lactobacilli, Gardnerella, and Mobiluncus, but it also adds the detection of Gram-positive cocci. Thus, differently to the Nugent score, it is useful also in the detection of aerobic vaginitis. The detection is semi-quantitative and more easily applied by the cytologist then the Nugent score.

Aerobic vaginitis can be detected exquisitely by wet mount microscopy. In fact, the scoring system by Donders (44) requires the detection of items such as leukocytes and parabasal cells which can only be found by microscopy (Table 5). Ancillary findings include increased pH and negative whiff test. The ISSVD and IUSTI/WHO guidelines agree on this, while the CDC and ACOG guidelines do not mention the condition. Although Gram staining microscopy can detect all the items for the Donders' score, it is currently not validated as a diagnostic tool for aerobic vaginitis, due to lack of dedicated criteria (27). Bacterial culturing and NAATs do not have a defined role in the diagnosis of aerobic vaginitis, although they may detect the bacterial species most involved in the specific case under analysis. Currently, there are no FDA approved NAAT platforms for the diagnosis of aerobic vaginitis.

**Table 5:** Donders’ score.

score	Lactobacillary grade	Leukocytes number	Toxic leukocytes	Background flora	Parabasal cells
0	I–IIa	≤10	None or sporadic	Unremarkable or cytolysis	<1% of total EC
1	IIb	>10/HPF - ≤ 10/EC	≤50% of total leukocytes	Small coliform bacilli	>1% and ≤10% of total EC
2	III	>10/EC	>50% of total leukocytes	Cocci or chains	>10% of total EC

This scoring system was developed by Donders to standardize the diagnosis of aerobic vaginitis (AV) (44). The analysis is performed with wet mount microscopy. It takes into account lactobacilli, leukocytes and background flora. HPF: high power field (400× magnification); EC, epithelial cell. The total score diagnoses: 0–2 Normal; 3–4: Mild AV; 5–6: Moderate AV; 7–10: Severe AV (desquamative inflammatory vaginitis—DIV).

Trichomoniasis can be detected by wet mount microscopy, Gram staining microscopy, appropriate culture, and NAATs. In wet mount microscopy, Trichomonas is easily recognizable for its motility. However, the motility is granted at body temperature, but this is inhibited with colder temperature, thus the sample must be examined immediately after collection. This advantage is lost with alcohol fixation and Gram staining, but the protozoal cells are still recognizable for their peculiar morphology, although their detection may be more difficult. Trichomonas culture has previously been the gold standard for diagnosis. However it is a complicated and time-consuming technique, but it has the advantage of revealing the microorganism even when present at low level. Similarly do NAATs, but more accurately and quickly. Thus, NAATs has become the new gold standard for the diagnosis of trichomoniasis according to all four guidelines mentioned, and several FDA approved platforms are available to diagnose this condition (114).

Cytolytic vaginosis is detectable only by microscopy, either wet mount microscopy or Gram staining microscopy or even with Papanicolau staining. Clearly, culturing or NAATs have no role in diagnosing cytolytic vaginosis. This is stated in the ISSVD guidelines (27), while the other three guidelines do not mention the condition. Typical microscopy findings for cytolytic vaginosis include bare nuclei, cytoplasm debris, abundant lactobacilli. The pH is always low, even below 4.0, typically 3.5–4.0. Whiff test is negative (81).

Sometimes, mixed conditions are found. For instance, the presence of abundancy of leukocytes in conditions that usually are not typically inflammatory, such as bacterial vaginosis or cytolytic vaginosis, should suggest the subtle presence of either Candida or Trichomonas, thus a yeast culture and a NAAT analysis for trichomoniasis are advisable (117–120).

## 7 Discussion

The choice of the correct therapy depends on the accurate diagnosis of the type of vaginitis. For instance, first line medications include azoles antifungals for candidiasis, metronidazole or clindamycin for bacterial vaginosis, oral 5-nitroimidazoles for trichomoniasis, clindamycin and/or hydrocortisone for aerobic vaginitis, and bicarbonate irrigations for cytolytic vaginosis (27–29, 121).

A 2020 study by Nyirjesy (122) conducted in the USA on 333 physicians and 984 patient charts showed that physicians were more aware of the diagnoses of candidiasis and bacterial vaginosis rather than the other types of vaginitis and often fell short on POC testing and adherence to guidelines on therapy. Even worse, in another study by Hillier (123) involving 303 women with vaginitis symptoms, provides additional evidence that clinical assessments frequently diverge from available guidelines, leading to many women with symptoms receiving inappropriate treatments. Thus, we must conclude that accurate diagnosis and treatment of vaginitis it is also a matter of appropriate medical education and practice.

Also, the availability of wide-spectrum medications, such as metronidazole-clotrimazole combination, lessen the urgency in many physicians for accurate diagnosis and may lead to empirical diagnostic approach to vaginitis. This medication may be effective for acute vulvovaginal candidiasis, episodic bacterial vaginosis, and trichomoniasis, although trichomoniasis requires a systemic/oral antibiotic therapy. Clearly, this drug combination is not effective for cytolytic vaginosis and has little influence on aerobic vaginitis. Moreover, mixed conditions can occur. Thus, this approach will not work effectively on a significant fraction of the patients complaining symptoms of vaginitis and many patients will recur shortly afterward (124–126).

This empirical “state of the art” suggests that some gynecologists and physicians treat various types of vaginitis as a singular condition, prescribing the same remedy for all cases. Fortunately, guidelines (27–30) have been developed to rigorously define each type of vaginitis and the appropriate treatments for them. While there is a clear need for quick diagnosis and timely treatment, the appropriateness of the intervention should not be overlooked. Therefore, the necessity for accurate and prompt POC testing is evident. Traditional POC tests include pH testing, “sniff/whiff” tests, and wet mount microscopy. By the use of these tests combined all types of vaginitis can be diagnosed (27). Besides directly detecting pathogens, wet mount microscopy is particularly useful to explore important details, such as inflammation and epithelial cells desquamation, that may affect symptoms (42).

One limitation of wet mount microscopy is that it requires appropriate instruments and training. Nowadays, microscopes are not considered part of the essential tool setting of the physician or gynecologist anymore (127). The advent of culturing and molecular testing has been a tide that washed away the need for owing a microscope and receiving appropriate education for its use. Powell (128), in her 2024 paper, argues that physicians should not resist with the traditional methods but, instead, they should embrace the new technologies and put them to appropriate use.

There may be good reasons for the gradual disuse of office wet mount microscopy. First, the microscope itself can be costly, and the required education for performing the exam is not readily available anymore (129). Also, the execution of wet mount microscopy may be time consuming, although well trained individuals would take only few minutes to complete the exam (129). Most importantly, wet mount microscopy resulted less sensitive when compared to laboratory tests such as culturing and NAATs, especially for candidiasis and trichomoniasis (130–133). Moreover, NAAT testing, which have overall great sensitivity and sensibility, have been increasingly available, also as POC testing, although more expensive than microscopy.

On the other hand, wet mount microscopy has proven as a quick, useful and inexpensive method for the diagnosis of vaginitis (37) and its accuracy can be effectively improved with appropriate training (129). Also, wet mount microscopy is the only validated method to diagnosing and scoring aerobic vaginitis and, along with Gram staining, cytolytic vaginosis (27). Finally, mixed conditions are probably better evaluated with wet mount microscopy rather than with other methods. Thus, although wet mount microscopy may not provide the highest accuracy in certain conditions, it is more reliable than relying solely on “history and physical examination” for adequately assessing the specific type of vaginitis and facilitating appropriate treatment. We must also consider that the limitations of wet mount microscopy mainly lie in its low sensitivity for detecting Candida or Trichomonas when they are present in low abundance. However, its specificity is high. Therefore, while a negative result is inconclusive, a positive result is diagnostic. The greatest advantages of wet mount microscopy compared to culturing or NAATs are that it allows for first-line screening to be completed in minutes and is cost-effective. For these reasons, it is an indispensable tool for the initial diagnosis of vaginitis.

In conclusion, wet mount microscopy is being dismissed from the routine gynecological visit too soon regardless its utility for the diagnosis of vaginitis (Table 6). Although less sensitive than culturing and NAATs, it provides a quick and reliable screening method that allows the clinician to promptly choose the most appropriate therapy in most cases (37). The microscopic evaluation may be completed by culturing or NAATs especially to rule out candidiasis or trichomoniasis, although the results will be available days later and require a follow up visit.

**Table 6:** Gold standards for the diagnosis of vaginitis.

Condition	Signs and symptoms	Wet mount microscopy	Gram staining microscopy	Culture	NAATs
Vulvovaginal candidiasis	Suggestive	Gold standard for IUSTI/WHO and CDC	Useful	Gold standard for ISSVD	Useful
Bacterial vaginosis	Suggestive	Useful	Gold standard	No role	No role
Aerobic vaginitis	Suggestive	Gold standard	Useful	No role	No role
Trichomoniasis	Not specific	Not sensitive	Not sensitive	Useful	Gold standard
Cytolytic vaginosis	Not specific	Gold standard	Useful	No role	No role

This table summarizes the indications from the ISSVD (27), CDC (30), ACOG (28), and IUSTI/WHO (29) guidelines. The gold standard methods for each type of vaginitis are shown according to guidelines. Other methods are commented based on actual relevance for clinical use, always according to guidelines.

With this paper, we would like to raise awareness among physicians and gynecologists on the most appropriate practice for diagnosing vaginitis and highlight the current role of office wet mount microscopy, which is currently neglected but it is still crucial for the accurate diagnosis of vaginitis.

## References

[1] Hildebrand JP Carlson K, Kansagor AT. Vaginitis—StatPearls—NCBI Bookshelf. Bethesda, MD: National Library of Medicine (2025). Available online at: https://www.ncbi.nlm.nih.gov/books/NBK470302/ (Accessed January 1, 2026).

[2] Paladine HL Desai UA. Vaginitis: diagnosis and treatment. Am Fam Physician. (2018) 97(5):321–9. 29671516

[3] Mills BB. Vaginitis beyond the basics. Obstet Gynecol Clin North Am. (2017) 44(2):159–77. 10.1016/j.ogc.2017.02.010 28499528

[4] Brown H Drexler M. Improving the diagnosis of vulvovaginitis: perspectives to align practice, guidelines, and awareness. Popul Heal Manag. (2020) 23(1_suppl):S3–12. 10.1089/pop.2020.0265 PMC7591372 32997581

[5] Ramírez-Santos A Pereiro M Toribio J. Recurrent vulvovaginitis: diagnostic assessment and therapeutic management. Actas Dermo-Sifiliográficas (Engl Ed). (2008) 99(3):190–8. 10.1016/s1578-2190(08)70231-2 18358194

[6] Goje O. Practical approach to recurrent vulvovaginitis. Contemp Ob Gyn. (2020) 65(04):1. Available online at: https://www.contemporaryobgyn.net/view/practical-approach-to-recurrent-vulvovaginitis (Accessed January 1, 2026).

[7] Grando D Watson CJ. Perspectives on vaginal ecology and management of recurrent vulvovaginal candidiasis: a narrative review. J Fungi. (2025) 11(11):806. 10.3390/jof11110806 PMC12653331 41295186

[8] Bradshaw CS Plummer EL Muzny CA Bacterial vaginosis. Nat Rev Dis Prim. (2025) 11(1):43. 10.1038/s41572-025-00626-1 40537474

[9] Galán JSJ Poliquin V Gerstein AC. Insights and advances in recurrent vulvovaginal candidiasis. PLOS Pathog. (2023) 19(11):e1011684. 10.1371/journal.ppat.1011684 37948448 PMC10637712

[10] Abbe C Mitchell CM. Bacterial vaginosis: a review of approaches to treatment and prevention. Front Reprod Heal. (2023) 5:1100029. 10.3389/frph.2023.1100029 PMC10264601 37325243

[11] Wu S Hugerth LW Schuppe-Koistinen I Du J. The right bug in the right place: opportunities for bacterial vaginosis treatment. NPJ Biofilms Microbiomes. (2022) 8(1):34. 10.1038/s41522-022-00295-y 35501321 PMC9061781

[12] Peddakolmi S Shiraskar O Bhor VM. Vaginal dysbiosis-associated infections: current and emerging treatment strategies. J Reprod Healthc Med. (2025) 6:23. 10.25259/jrhm_32_2025

[13] Riaz R Khan K Aghayeva S Uddin R. Combatting antibiotic resistance in Gardnerella vaginalis: a comparative in silico investigation for drug target identification. PLoS One. (2025) 20(3):e0314465. 10.1371/journal.pone.0314465 40073044 PMC12372832

[14] Bautista CT Wurapa EK Sateren WB Morris SM Hollingsworth BP Sanchez JL. Association of bacterial vaginosis with chlamydia and gonorrhea among women in the U.S. army. Am J Prev Med. (2017) 52(5):632–9. 10.1016/j.amepre.2016.09.016 27816380

[15] Lovett A Seña AC Macintyre AN Sempowski GD Duncan JA Waltmann A. Cervicovaginal microbiota predicts neisseria gonorrhoeae clinical presentation. Front Microbiol. (2022) 12:790531. 10.3389/fmicb.2021.790531 35222300 PMC8867028

[16] Tchernodrinski TS Ma W Sato A Goba GK Shi E Kuang M. Correlation of sexually transmitted infections and bacterial vaginosis recurrence [ID 747]. Obstet Gynecol. (2025) 145(6S):20S–20. 10.1097/aog.0000000000005916.065

[17] Nava-Memije K Hernández-Cortez C Ruiz-González V Bacterial vaginosis and sexually transmitted infections in an HIV-positive cohort. Front Reprod Heal. (2021) 3:660672. 10.3389/frph.2021.660672 PMC9580688 36303986

[18] Byun JM Jeong DH Kim YN Differences in sexually transmitted infection-associated cervical infections in pelvic inflammatory disease patients between adolescents and adults. Taiwan J Obstet Gynecol. (2025) 64(2):265–71. 10.1016/j.tjog.2024.10.015 40049810

[19] Cohen CR Lingappa JR Baeten JM Bacterial vaginosis associated with increased risk of female-to-male HIV-1 transmission: a prospective cohort analysis among African couples. PLoS Med. (2012) 9(6):e1001251. 10.1371/journal.pmed.1001251 22745608 PMC3383741

[20] Asare K Ngcapu S Osman F Incidence, recurrence, and prevalence of bacterial vaginosis from acute to chronic HIV infection in a prospective cohort of women in South Africa. Ann Epidemiol. (2023) 82:33–9. 10.1016/j.annepidem.2023.04.004 37037344 PMC10247472

[21] Mtshali A Ngcapu S Mindel A Garrett N Liebenberg L. HIV susceptibility in women: the roles of genital inflammation, sexually transmitted infections and the genital microbiome. J Reprod Immunol. (2021) 145:103291. 10.1016/j.jri.2021.103291 33647576

[22] Martins BCT Guimarães RA Alves RRF Saddi VA. Bacterial vaginosis and cervical human papillomavirus infection in young and adult women: a systematic review and meta-analysis. Rev Saúde Pública. (2022) 56:113. 10.11606/s1518-8787.2022056004412 PMC9749738 36629704

[23] Qi J Dai C Song L Zhang J. Association between bacterial vaginosis with human papillomavirus in the United States (NHANES 2003–2004). BMC Women’s Heal. (2024) 24(1):138. 10.1186/s12905-024-02956-w PMC10882805 38388384

[24] Mohanty T Doke PP Khuroo SR. Effect of bacterial vaginosis on preterm birth: a meta-analysis. Arch Gynecol Obstet. (2023) 308(4):1247–55. 10.1007/s00404-022-06817-5 36251068

[25] Hadhoum S Subtil D Labreuche J Reassessing the association between bacterial vaginosis and preterm birth: a systematic review and meta-analysis. J Gynecol Obstet Hum Reprod. (2025) 54(1):102871. 10.1016/j.jogoh.2024.102871 39442804

[26] Geer K Klega A. Vaginitis: diagnosis and treatment. Am Fam Physician. (2025) 112(5):504–12. 41252833

[27] International Society for the Study of Vulvovaginal Disease Recommendations for the Diagnosis and Treatment of Vaginitis. (2023). *Published online*. 10.59153/adm.rdtv.001

[28] The American College of Obstetricians and Gynecologists. Vaginitis in nonpregnant patients: aCOG practice bulletin, number 215. Obstet Gynecol. (2020) 135(1):e1–17. 10.1097/aog.0000000000003604 31856123

[29] Sherrard J Wilson J Donders G Mendling W Jensen JS. 2018 European (IUSTI/WHO) international union against sexually transmitted infections (IUSTI) world health organisation (WHO) guideline on the management of vaginal discharge. Int J STD AIDS. (2018) 29(13):1258–72. 10.1177/0956462418785451 30049258

[30] Workowski KA Bachmann LH Chan PA Sexually transmitted infections treatment guidelines, 2021. MMWR Recomm Rep. (2021) 70(4):1–187. 10.15585/mmwr.rr7004a1 PMC8344968 34292926

[31] Khalilzadeh S Eftekhar T Rahimi R Mehriardestani M Tabarrai M. An evidence-based review of medicinal plants used for the treatment of vaginitis by avicenna in “the canon of medicine. Galen Méd J. (2019) 8(0):1270. 10.31661/gmj.v8i0.1270 PMC8344152 34466482

[32] Zhou J Qu F. Treating gynaecological disorders with traditional Chinese medicine: a review. Afr J Tradit Complement Altern Med. (2009) 6(4):494–517. 10.4314/ajtcam.v6i4.57181 20606770 PMC2816470

[33] Davis IM. Antoni van Leeuwenhoek and measuring the invisible: the context of 16th and 17th century micrometry. Stud Hist Philos Sci Part A. (2020) 83:75–85. 10.1016/j.shpsa.2020.03.004 32958283

[34] Margolis E Fredricks DN. Part 13: Sexually Transmitted Infections, Bacterial vaginosis-associated bacteria. In: Tang Y-W Liu D Sussman M Poxton I Schwartzman J, editors. Molecular Medical Microbiology. 2nd ed. New York: Academic Press (2015). p. 1487–96. 10.1016/b978-0-12-397169-2.00083-4

[35] Chacra LA Fenollar F Diop K. Bacterial vaginosis: what do we currently know? Front Cell Infect Microbiol. (2022) 11:672429. 10.3389/fcimb.2021.672429 35118003 PMC8805710

[36] Brusselmans J Sutter AD Devleesschauwer B Verstraelen H Cools P. Scoping review of the association between bacterial vaginosis and emotional, sexual and social health. BMC Women’s Heal. (2023) 23(1):168. 10.1186/s12905-023-02260-z PMC10080849 37029382

[37] Vieira-Baptista P Grincevičienė Š Oliveira C Fonseca-Moutinho J Stockdale CK Chery F. The international society for the study of vulvovaginal disease vaginal wet mount microscopy guidelines: how to perform, applications, and interpretation. J Low Genit Tract Dis. (2021) 25(2):172–80. 10.1097/lgt.0000000000000595 33631782

[38] Srb N Talapko J Meštrović T A comprehensive overview of Candida albicans as the leading pathogen in vulvovaginal candidiasis. J Fungi. (2025) 11(9):632. 10.3390/jof11090632 PMC12471164 41003178

[39] Sustr V Foessleitner P Kiss H Farr A. Vulvovaginal candidosis: current concepts, challenges and perspectives. J Fungi. (2020) 6(4):267. 10.3390/jof6040267 PMC7712750 33171784

[40] Bhosale VB Koparde AA Thorat VM. Vulvovaginal candidiasis-an overview of current trends and the latest treatment strategies. Microb Pathog. (2025) 200:107359. 10.1016/j.micpath.2025.107359 39921042

[41] Sonnex C Lefort W. Microscopic features of vaginal candidiasis and their relation to symptomatology. Sex Transm Infect. (1999) 75(6):417. 10.1136/sti.75.6.417 10754949 PMC1758262

[42] Mylonas I Bergauer F. Diagnosis of vaginal discharge by wet mount microscopy & colon; a simple and underrated method. Obstet Gynecol Surv. (2011) 66(6):359–68. 10.1097/ogx.0b013e31822bdf31 21851750

[43] Donders GGG Bellen G Grinceviciene S Ruban K Vieira-Baptista P. Aerobic vaginitis: no longer a stranger. Res Microbiol. (2017) 168(9–10):845–58. 10.1016/j.resmic.2017.04.004 28502874

[44] Donders GGG Vereecken A Bosmans E Dekeersmaecker A Salembier G Spitz B. Definition of a type of abnormal vaginal flora that is distinct from bacterial vaginosis: aerobic vaginitis. BJOG. (2002) 109(1):34–43. 10.1111/j.1471-0528.2002.00432.x 11845812

[45] Tian W Li Y Zhang Y Systematic review and meta-analysis of the global prevalence and infection risk factors of trichomonas vaginalis. Parasite. (2025) 32:56. 10.1051/parasite/2025051 40864904 PMC12386857

[46] Schumann JA Plasner S. Trichomoniasis. In: StatPearls. Treasure Island, FL: StatPearls Publishing (2026). Available online at: https://www.ncbi.nlm.nih.gov/books/NBK534826/ (Accessed January 1, 2026).

[47] Patil MJ Nagamoti JM Metgud SC. Diagnosis of trichomonas vaginalis from vaginal specimens by wet mount microscopy, in pouch TV culture system, and PCR. J Glob Infect Dis. (2012) 4(1):22–25. 10.4103/0974-777x.93756 22529623 PMC3326953

[48] Suresh A Rajesh A Bhat RM Rai Y. Cytolytic vaginosis: a review. Indian J Sex Transm Dis AIDS. (2009) 30(1):48–50. 10.4103/2589-0557.55490 21938117 PMC3168042

[49] Kraut R Carvallo FD Golonka R Scoping review of cytolytic vaginosis literature. PLoS One. (2023) 18(1):e0280954. 10.1371/journal.pone.0280954 36701339 PMC9879469

[50] Varma K Kansal M. Cytolytic vaginosis: a brief review. J Ski Sex Transm Dis. (2022) 4(2):206–10. 10.25259/jsstd_41_2021

[51] Ventolini G Gandhi K Manales NJ Garza J Sanchez A Martinez B. Challenging vaginal discharge, lactobacillosis and cytolytic vaginitis. J Fam Reprod Heal. (2022) 16(2):102–5. 10.18502/jfrh.v16i2.9477 PMC9678848 36457655

[52] Stika CS. Atrophic vaginitis. Dermatol Ther. (2010) 23(5):514–22. 10.1111/j.1529-8019.2010.01354.x 20868405

[53] Donders GGG Donders FHWV. New developments in the management of vulvovaginal atrophy: a comprehensive overview. Expert Opin Pharmacother. (2023) 24(5):599–616. 10.1080/14656566.2023.2194017 36951262

[54] Sweitzer S Duncan JA Seña AC. Update on syphilis diagnostics. Curr Opin Infect Dis. (2025) 38(1):44–53. 10.1097/qco.0000000000001073 39641765 PMC11695141

[55] Cao W Thorpe PG O’Callaghan K Kersh EN. Advantages and limitations of current diagnostic laboratory approaches in syphilis and congenital syphilis. Expert Rev Anti-Infect Ther. (2023) 21(12):1339–54. 10.1080/14787210.2023.2280214 37934903 PMC10958575

[56] Vendhan S Vasudevan B Bala K Neema S. Enhancing syphilis diagnosis through innovative adaptation of wet mount microscopy. Indian J Dermatol Venereol Leprol. (2024) 90(6):844–5. 10.25259/ijdvl_374_2024 39152810

[57] Cai S Pan J Duan D Yu C Yang Z Zou J. Prevalence of ureaplasma urealyticum, chlamydia trachomatis, and Neisseria gonorrhoeae in gynecological outpatients, Taizhou, China. J Clin Lab Anal. (2020) 34(2):e23072. 10.1002/jcla.23072 31675147 PMC7031556

[58] He M Xie Y Zhang R Prevalence and antimicrobial resistance of mycoplasmas and chlamydiae in patients with genital tract infections in Shanghai, China. J Infect Chemother. (2016) 22(8):548–52. 10.1016/j.jiac.2016.05.007 27324895

[59] Randjelovic I Moghaddam A Blasio BF de Moi H. The role of polymorphonuclear leukocyte counts from urethra, cervix, and vaginal wet mount in diagnosis of nongonococcal lower genital tract infection. Infect Dis Obstet Gynecol. (2018) 2018(1):8236575. 10.1155/2018/8236575 30147292 PMC6083538

[60] Raphaelidis L. Uncommon vaginitis cases: expect the unexpected. J Nurse Pr. (2015) 11(1):135–8. 10.1016/j.nurpra.2014.07.037

[61] Wang M Gao Z. Sexual dysfunction in women with Sjögren’s syndrome: a cross-sectional observational study. J Obstet Gynaecol. (2025) 45(1):2463413. 10.1080/01443615.2025.2463413 39945612

[62] Oliveira AS Rolo J Gaspar C Oliveira RP de Oliveira JM de Oliveira AP de. Allergic vulvovaginitis: a systematic literature review. Arch Gynecol Obstet. (2022) 306(3):593–622. 10.1007/s00404-021-06332-z 34825938

[63] Diamantis A Magiorkinis E Androutsos G. Alfred Françcois Donné (1801–78): a pioneer of microscopy, microbiology and haematology. J Méd Biogr. (2009) 17(2):81–7. 10.1258/jmb.2008.008040 19401511

[64] O’Sullivan JF. Trichomonas vaginalis. Ir J Méd Sci (1926–1967). (2008) 42(5):207. 10.1007/bf02954274 4859730

[65] World Health Organization. Recommendations for the treatment of trichomonas vaginalis, mycoplasma genitalium, Candida albicans, bacterial vaginosis and human papillomavirus (anogenital warts): Web annex D: evidence-to-decision framework and systematic review for the WHO treatment recommendations for bacterial vaginosis. (2024):401–11. Available online at: https://iris.who.int/handle/10665/378219 (Accessed January 1, 2026). 39116264

[66] Wilkinson JS. Some remarks upon the development of epiphytes. With the description of a new vegetable formation found in connexion with the human uterus. Lancet. (1849) 54(1365):448–51. 10.1016/s0140-6736(00)63203-8

[67] Barnett JA. A history of research on yeasts 8: taxonomy. Yeast. (2004) 21(14):1141–93. 10.1002/yea.1154 15515119

[68] Ligon BL. Albert Ludwig Sigesmund Neisser: discoverer of the cause of gonorrhea. Semin Pediatr Infect Dis. (2005) 16(4):336–41. 10.1053/j.spid.2005.07.001 16210113

[69] Kirkcaldy RD Weston E Segurado AC Hughes G. Epidemiology of gonorrhoea: a global perspective. Sex Heal. (2019) 16(5):401–411. 10.1071/sh19061 PMC7064409 31505159

[70] David M. Albert und Gustav Döderlein—ein kritischer Blick auf zwei besondere Lebensläufe deutscher Ordinarien [Albert and Gustav Döderlein—a critical view to the biographies of two German professors]. Zentralbl Gynakol. (2006) 128(2):56–9. German. 10.1055/s-2006-921412 16673245

[71] Doderlein A. Das Scheidensekret Und Seine Bedeutung Fur Das Puerperalfieber. Vol 1. Leipzig: Kessinger Publishing (1892).

[72] Waugh M. The centenary of Treponema pallidum: on the discovery of spirochaeta pallida. Int J STD AIDS. (2005) 16(9):594–5. 10.1258/0956462054944534 16176623

[73] Zhu X Zhang W Fei J Zhou J. Cervical syphilitic lesions mimicking cervical cancer: a rare case report. Int J Infect Dis. (2015) 31:1–3. 10.1016/j.ijid.2014.12.005 25486010

[74] Yu W You X Luo W. Global, regional, and national burden of syphilis, 1990–2021 and predictions by Bayesian age-period-cohort analysis: a systematic analysis for the global burden of disease study 2021. Front Med. (2024) 11:1448841. 10.3389/fmed.2024.1448841 PMC11357943 39211337

[75] Gardner HL Dukes CD. Haemophilus vaginalis vaginitis: a newly defined specific infection previously classified non-specific vaginitis. Am J Obstet Gynecol. (1955) 69(5):962–76. 14361525

[76] Gardner HL Dukes CD. Hemophilus vaginalis vaginitis. Ann N York Acad Sci. (1959) 83(2):280–9. 10.1111/j.1749-6632.1960.tb40901.x 13826525

[77] Piot P Dyck EV Goodfellow M Falkow S. A taxonomic study of Gardnerella vaginalis (Haemophilus vaginalis) gardner and dukes 1955. Microbiology. (1980) 119(2):373–96. 10.1099/00221287-119-2-373 6971916

[78] Spiegel CA Davick P Totten PA Gardnerella vaginalis and anaerobic bacteria in the etiology of bacterial (nonspecific) vaginosis. Scand J Infect Dis Suppl. (1983) 40:41–46. 6607521

[79] Amsel R Totten PA Spiegel CA Chen KCS Eschenbach D Holmes KK. Nonspecific vaginitis diagnostic criteria and microbial and epidemiologic associations. Am J Med. (1983) 74(1):14–22. 10.1016/0002-9343(83)91112-9 6600371

[80] Cibley LJ Cibley LJ. Cytolytic vaginosis. Am J Obstet Gynecol. (1991) 165(4):1245–9. 10.1016/s0002-9378(12)90736-x 1951582

[81] Kömeç S Tercan C Ceylan AN Durmuş MA Donbaloğlu GŞ Aydın MD. Cytolytic vaginosis in women with vaginitis: prevalence, diagnosis, and treatment. Gynecol Obstet Investig. 2025:1–7. Published online. 10.1159/000548768 41045474

[82] Coico R. Gram staining. Curr Protoc Microbiol. (2006) 00(1):A.3C.1–2. 10.1002/9780471729259.mca03cs00 18770544

[83] Barcia JJ. The Giemsa stain: its history and applications. Int J Surg Pathol. (2007) 15(3):292–6. 10.1177/1066896907302239 17652540

[84] Kootallur B Thangavelu C Mani M. Bacterial identification in the diagnostic laboratory: how much is enough? Indian J Méd Microbiol. (2011) 29(4):336–40. 10.4103/0255-0857.90156 22120791

[85] Gajic I Kabic J Kekic D Antimicrobial susceptibility testing: a comprehensive review of currently used methods. Antibiotics. (2022) 11(4):427. 10.3390/antibiotics11040427 35453179 PMC9024665

[86] Stewart EJ. Growing unculturable bacteria. J Bacteriol. (2012) 194(16):4151–60. 10.1128/jb.00345-12 22661685 PMC3416243

[87] Sheeley A. Sorting out common causes of abnormal vaginal discharge. JAAPA. (2004) 17(10):15–6, 18–20, 22. 15532320

[88] Caillouette JC Sharp CF Zimmerman GJ Roy S. Vaginal pH as a marker for bacterial pathogens and menopausal status. Am J Obstet Gynecol. (1997) 176(6):1270–7. 10.1016/s0002-9378(97)70345-4 9215184

[89] Hellberg D Nilsson S Mårdh PA. The diagnosis of bacterial vaginosis and vaginal flora changes. Arch Gynecol Obstet. (2001) 265(1):11–5. 10.1007/s004040000109 11327086

[90] Nugent RP Krohn MA Hillier SL. Reliability of diagnosing bacterial vaginosis is improved by a standardized method of gram stain interpretation. J Clin Microbiol. (1991) 29(2):297–301. 10.1128/jcm.29.2.297-301.1991 1706728 PMC269757

[91] Bansal R Garg P Garg A. Comparison of Amsel’s criteria and Nugent’s criteria for diagnosis of bacterial vaginosis in tertiary care centre. Int J Reprod Contracept Obstet Gynecol. (2018) 8(2):637–40. 10.18203/2320-1770.ijrcog20190297

[92] Srinivasan U Ponnaluri S Villareal L Gram stains: a resource for retrospective analysis of bacterial pathogens in clinical studies. PLoS One. (2012) 7(10):e42898. 10.1371/journal.pone.0042898 23071487 PMC3469605

[93] Tanaka T Kagemoto K Hirata K. Predicting yeast-like fungi from gram stained smears images. Proc 2024 7th Int Conf Digit Med Image Process. (2024):84–87. Published online. 10.1145/3705927.3705942

[94] Magliano E. The microbiological diagnosis of Chlamydia trachomatis infections: milestones from a centenary history. Microbiol Med. (2016) 31(2):63–5. 10.4081/mm.2016.6103

[95] Eldin C Parola P Raoult D. Limitations of diagnostic tests for bacterial infections. Médecine Mal Infect. (2019) 49(2):98–101. 10.1016/j.medmal.2018.12.004 30686500

[96] Shrier LA Dean D Klein E Harter K Rice PA. Limitations of screening tests for the detection of chlamydia trachomatis in asymptomatic adolescent and young adult women. Am J Obstet Gynecol. (2004) 190(3):654–62. 10.1016/j.ajog.2003.09.063 15041995

[97] Frølund M Björnelius E Lidbrink P Ahrens P Jensen JS. Comparison between culture and a multiplex quantitative real-time polymerase chain reaction assay detecting ureaplasma urealyticum and U. parvum. PLoS One. (2014) 9(7):e102743. 10.1371/journal.pone.0102743 25047036 PMC4105565

[98] Serena TE Bowler PG Schultz GS D’souza Rennie YM. Are semi-quantitative clinical cultures inadequate? Comparison to quantitative analysis of 1053 bacterial isolates from 350 wounds. Diagnostics. (2021) 11(7):1239. 10.3390/diagnostics11071239 34359322 PMC8303231

[99] Fredricks DN Fiedler TL Thomas KK Oakley BB Marrazzo JM. Targeted PCR for detection of vaginal bacteria associated with bacterial vaginosis. J Clin Microbiol. (2007) 45(10):3270–6. 10.1128/jcm.01272-07 17687006 PMC2045326

[100] Amor I Alberola A Salazar AD Evaluation of the vaginal panel realtime PCR kit (vircell, SL) for diagnosing vaginitis: a comparative study with routinely used diagnostics. PLoS One. (2024) 19(11):e0313414. 10.1371/journal.pone.0313414 39504318 PMC11540222

[101] Navarathna DH Lukey J Coppin JD Jinadatha C. Diagnostic performance of DNA probe-based and PCR-based molecular vaginitis testing. Microbiol Spectr. (2023) 11(5):e01628–23. 10.1128/spectrum.01628-23 37615484 PMC10581173

[102] Breding K Vikström I Selbing A Farnebäck M Hermelin A Larsson PG. Diagnosis of bacterial vaginosis using a novel molecular real-time PCR test. JWHG. (2020) 7:1–7. 10.17303/jwhg.2020.7.102

[103] Berman H McLaren M Callahan B. Understanding and interpreting community sequencing measurements of the vaginal microbiome. BJOG. (2020) 127(2):139–46. 10.1111/1471-0528.15978 31597208 PMC10801814

[104] Liu S Chen Y Zhang K Exploring vaginal microbiome: from traditional methods to metagenomic next-generation sequencing—a systematic review. Front Microbiol. (2025) 16:1578681. 10.3389/fmicb.2025.1578681 40895484 PMC12391137

[105] Wied GL. Phase-contrast microscopy, an office technique for prescreening of cytologic vaginal smears. Am J Obstet Gynecol. (1956) 71(4):806–18. 10.1016/0002-9378(56)90577-4 13302317

[106] Kelly KG. Chapter 179 Tests on vaginal discharge. In: Walker HK Hall WD Hurst JW, editors. Clinical Methods: The History, Physical, and Laboratory Examinations. 3rd ed. Boston: Butterworths (1990) p. 833–6. Available online at: https://www.ncbi.nlm.nih.gov/books/NBK288/ (Accessed January 1, 2026). 21250045

[107] Donders GGG Vereecken A Dekeersmaecker A Bulck BV Spitz B. Wet mount microscopy reflects functional vaginal lactobacillary flora better than gram stain. J Clin Pathol. (2000) 53(4):308. 10.1136/jcp.53.4.308 10823128 PMC1731171

[108] Schmidt H Hansen JG. Validity of wet-mount bacterial morphotype identification of vaginal fluid by phase-contrast microscopy for diagnosis of bacterial vaginosis in family practice note. APMIS. (2001) 109(9):589–94. 10.1034/j.1600-0463.2001.d01-179.x 11878711

[109] Hillier SL Critchlow CW Stevens CE Microbiological, epidemiological and clinical correlates of vaginal colonisation by mobiluncus species. Genitourin Med. (1991) 67(1):26. 10.1136/sti.67.1.26 1916772 PMC1194609

[110] Ghanbarzade N Moghanni M Goljahani N Karamian M Bidaki MZ. Frequency of Neisseria gonorrhoeae and trichomonas vaginalis in women with vaginal discharge referring to the gynecology clinic of vali-e-asr hospital in birjand in 2018. J Basic Res Med Sci. (2019). Published online.

[111] Kesli R Pektas B Ozdemir M Microscopic examination of vaginal discharge specimens for trichomonas vaginalis and other micro-organisms in 18–45 age group women. Turk J Parasitol. (2012) 36(3):182–4. 10.5152/tpd.2012.43 23169165

[112] Knoll MA Steixner S Lass-Flörl C. How to use direct microscopy for diagnosing fungal infections. Clin Microbiol Infect. (2023) 29(8):1031–8. 10.1016/j.cmi.2023.05.012 37187349

[113] Donders GGG Larsson PG Platz-Christensen JJ Hallén A Meijden W van der Wölner-Hanssen P. Variability in diagnosis of clue cells, lactobacillary grading and white blood cells in vaginal wet smears with conventional bright light and phase contrast microscopy. Eur J Obstet Gynecol Reprod Biol. (2009) 145(1):109–12. 10.1016/j.ejogrb.2009.04.012 19481329

[114] Nucleic Acid Based Tests | FDA. (2026). Available online at: https://www.fda.gov/medical-devices/*in-vitro*-diagnostics/nucleic-acid-based-tests (Accessed January 1, 2026).

[115] Narasimhan V Kim H Lee SH Nucleic acid amplification-based technologies (NAAT)—toward accessible, autonomous, and Mobile diagnostics. Adv Mater Technol. (2023) 8(20). 10.1002/admt.202300230

[116] Ison CA Hay PE. Validation of a simplified grading of gram stained vaginal smears for use in genitourinary medicine clinics. Sex Transm Infect. (2002) 78(6):413. 10.1136/sti.78.6.413 12473800 PMC1758337

[117] Qi W Li H Wang C Recent advances in presentation, diagnosis and treatment for mixed vaginitis. Front Cell Infect Microbiol. (2021) 11:759795. 10.3389/fcimb.2021.759795 34796129 PMC8592905

[118] Said SK Ishimwe MPS Kasujja M Mixed vaginal infections and their predictors among women with abnormal vaginal discharges attending gynecological clinics in western Uganda: a cross-sectional study. Interdiscip Perspect Infect Dis. (2025) 2025(1):6511013. 10.1155/ipid/6511013 40918419 PMC12411039

[119] Jun L Wan X Zhang D Mixed vaginal infection status in women infected with trichomonas vaginalis: comparison of microscopy method and metagenomic sequencing analysis. Front Cell Infect Microbiol. (2025) 15:1638464. 10.3389/fcimb.2025.1638464 41416110 PMC12708925

[120] Benyas D Sobel JD. Mixed vaginitis due to bacterial vaginosis and candidiasis. J Low Genit Tract Dis. (2022) 26(1):68–70. 10.1097/lgt.0000000000000641 34840242

[121] Hazra A Collison MW Davis AM. CDC sexually transmitted infections treatment guidelines, 2021. JAMA. (2022) 327(9):870–1. 10.1001/jama.2022.1246 35230409

[122] Nyirjesy P Banker WM Bonus TM. Physician awareness and adherence to clinical practice guidelines in the diagnosis of vaginitis patients: a retrospective chart review. Popul Heal Manag. (2020) 23(1_suppl):S13–21. 10.1089/pop.2020.0258 PMC7591374 32985960

[123] Hillier SL Austin M Macio I Meyn LA Badway D Beigi R. Diagnosis and treatment of vaginal discharge syndromes in community practice settings. Clin Infect Dis. (2020) 72(9):1538–43. 10.1093/cid/ciaa260 PMC8248297 32350529

[124] Huang Y Shen C Shen Y Cui H. Assessing the efficacy of clotrimazole and metronidazole combined treatment in vaginitis: a meta-analysis. Altern Ther Heal Med. (2024) 30(1):186–91. 37773671

[125] Huang SH Hsu HC Lee TF Prevalence, associated factors, and appropriateness of empirical treatment of trichomoniasis, bacterial vaginosis, and vulvovaginal candidiasis among women with vaginitis. Microbiol Spectr. (2023) 11(3):e00161–23. 10.1128/spectrum.00161-23 37052487 PMC10269550

[126] Sobel JD. Syndromic treatment of women with vulvovaginal symptoms in the United States: a call to action!. Clin Infect Dis. (2020) 72(9):1544–5. 10.1093/cid/ciaa267 32350527

[127] Faro S. What has happened to the microscope in evaluating obstetric and gynecologic patients? Obstet Gynecol Int J. (2022) 13(3):172–2. 10.15406/ogij.2022.13.00645

[128] Powell A Goje O Nyirjesy P. A comparison of newer and traditional approaches to diagnosing vaginal infections. Obstet Gynecol. (2024) 143(4):491–8. 10.1097/aog.0000000000005529 38350107

[129] Donders GGG Marconi C Bellen G Donders F Michiels T. Effect of short training on vaginal fluid microscopy (wet mount). Learning. J Low Genit Tract Dis. (2015) 19(2):165–9. 10.1097/lgt.0000000000000052 25148226

[130] Nabweyambo S Kakaire O Sowinski S Very low sensitivity of wet mount microscopy compared to PCR against culture in the diagnosis of vaginal trichomoniasis in Uganda: a cross sectional study. BMC Res Notes. (2017) 10(1):259. 10.1186/s13104-017-2581-1 28683790 PMC5501264

[131] Roy P Mirza TT Paul SK Comparison of wet mount microscopy and Giemsa staining to PCR in the diagnosis of vaginal trichomoniasis in a tertiary level hospital of Bangladesh. Mymensingh Méd J: MMJ. (2023) 32(2):348–54. 37002744

[132] El-Karseem NMA Dyab AK Albalawi NO Microscopic and molecular detection of trichomonas vaginalis in outpatients seeking medical care in upper Egypt. Front Microbiol. (2024) 15:1499270. 10.3389/fmicb.2024.1499270 39633806 PMC11615069

[133] Danby CS Althouse AD Hillier SL Wiesenfeld HC. Nucleic acid amplification testing compared with cultures, gram stain, and microscopy in the diagnosis of vaginitis. J Low Genit Tract Dis. (2021) 25(1):76–80. 10.1097/lgt.0000000000000576 33347046

